# Protecting Physical Communications in 5G C-RAN Architectures through Resonant Mechanisms in Optical Media

**DOI:** 10.3390/s20154104

**Published:** 2020-07-23

**Authors:** Borja Bordel Sánchez, Ramón Alcarria, Tomás Robles, Antonio Jara

**Affiliations:** 1 Escuela Politécnica Superior, Universidad Alfonso X el Sabio, UAX, Avenida Universidad, 1, Villanueva de la Cañada, 28691 Madrid, Spain; 2 Department of Geospatial Engineering, Universidad Politécnica de Madrid, UPM Campus Sur, Km 7.5 de la Autovía de Valencia, 28031 Madrid, Spain; ramon.alcarria@upm.es; 3 Department of Information Systems, Universidad Politécnica de Madrid, UPM Campus Sur, Km 7.5 de la Autovía de Valencia, 28031 Madrid, Spain; tomas.robles@upm.es; 4 Institute of Information Systems, University of Applied Sciences Western Switzerland (HES-SO), Techno-Pôle 3, 3960 Sierre, Valais, Switzerland; jara@ieee.org

**Keywords:** 5G networks, steganography, chaotic encryption, resonant optical structures, C-RAN architecture

## Abstract

Future 5G networks are characterized by three basic ideas: enhanced mobile broadband communications, massive machine-type communications, and ultra-low-latency communications. Any of these requirements needs, to be fulfilled, the implementation of high-efficiency technologies at all levels. This includes some of the costliest mechanisms in terms of computational time and bitrate: information protection solutions. Typical techniques in this area employ complex algorithms and large protocol headers, which strongly reduces the effective baud rate and latency of future 5G networks and communications. This is especially relevant in the access network, which in 5G networks will follow a cloud-based architecture, where thousands of different devices must communicate, before aggregating all those streams to be sent to the backbone. Then, new and more efficient mechanisms are needed in the cloud radio access networks (C-RAN) for future 5G systems. Therefore, in this paper it is proposed a novel information protection scheme for C-RAN architectures based on resonant phenomena in optical fibers communicating the fronthaul and backhaul in 5G networks. Resonant structures and physical nonlinearities generate a chaotic signal which may encrypt and hide at physical level every communication stream in a very efficient manner. To evaluate the proposed mechanism, an experimental validation based on simulation techniques is also described and results discussed.

## 1. Introduction

Many different architectures and paradigms have been proposed as an optimal solution for implementing future 5G networks [1]. From multi-frequency schemes combining microcells and macrocells [2]; to virtual infrastructures managed through commercial mechanisms such as Kubernettes [3]. Any case, all authors agree any solution for future 5G networks must guarantee three basic characteristics. Namely:

- Enhanced Mobile Broadband Communications (eMBC) [4]: Mobile nodes and devices must be provided with very high-speed communication links. Typically, experts indicate that effective bitrate should be above 50 Mbps.
- Ultra-Reliable Low Latency Communications (URLLC) [5]: Communication links supported by 5G networks must guarantee a very low delay and jitter in transmissions with remote nodes. According to most authors, jitter must be null and communication delays be below 10 (ten) milliseconds.
- Massive Machine-Type Communications (MMTC) [6]: In future 5G networks thousands of nodes and devices in pervasive infrastructures and dense deployments must communicate concurrently. All of them must be provided with eMBC and URLLC, and mobile networks must be able to handle these scenarios.

More efficient algorithms, communication protocols and network architectures are needed to meet these requirements. However, before all these engineering considerations, physical media connecting machines to be communicated must have a capacity high enough to support eMBC, URLLC and MMTC, all together and at the same time [7].

**Figure 1:**
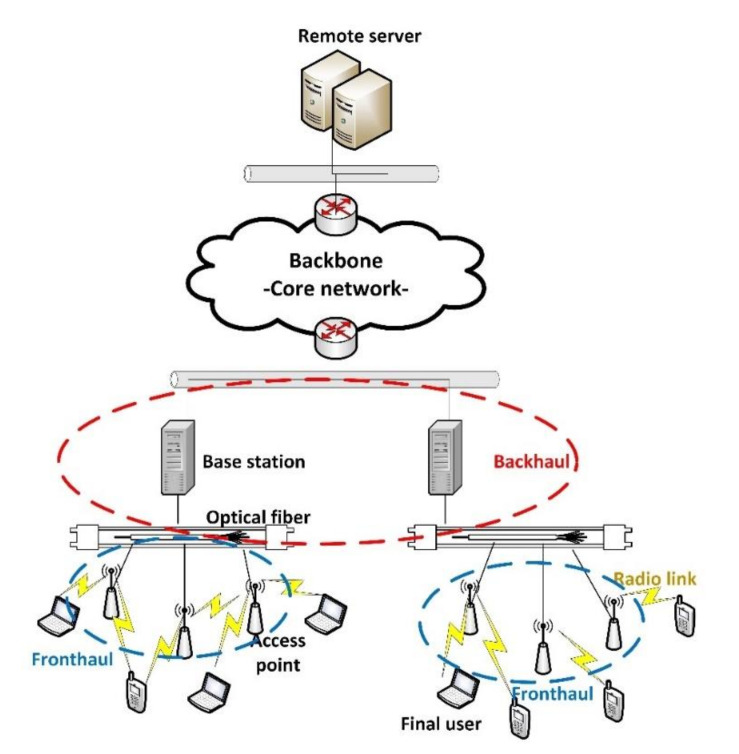
Scheme of C-RAN architectures in future 5G networks.

Nowadays, wireless links present much higher latencies and lower capacity than most modern wired transmission mechanisms [8], specifically than modern optical fiber technologies. Nevertheless, wireless channels are essential to guarantee mobility, the basic characteristic of mobile networks. Thus, in future 5G networks (as in novel 4G+ network deployments), physical media are following a hybrid approach [9]. A large number of wireless radio access points are placed close to final users, so transmission delays in those channels go down at the same time their physical capacity goes up (as the effective radio Signal-to-Noise ratio is higher). Besides, as several radio access points are deployed, it is easier to manage dense environments and pervasive infrastructures. Then, all these radio access points (and radio links) are communicated through high capacity optical fiber links to the base station, where data are processed and managed. This access network, then, communicates with the core network using traditional TCP/IP or Multiprotocol Label Switching (MPLS) solutions. The resulting physical infrastructure is usually known as Cloud Radio Access Network or C-RAN [10] (see Figure 1).

C-RAN infrastructures are, in general words, composed of a fronthaul network (made of proximity radio access points), and a backhaul network, where full capacity base stations are included. Both networks are communicated through high performance optical links. Final users get connected to the fronthaul using radio mechanisms, which must be also adapted to meet the requirement of 5G networks. The backhaul may communicate to the backbone (or core network) using efficient packet communication technologies such as MPLS.

Access points in the 5G fronthaul are designed to forward messages in a very fast and efficient manner, as they have limited decision and processing capabilities (which are mainly deployed in base stations). Then, every solution deployed together with these access points in the fronthaul must fulfill these principia. In this context, one of the most critical mechanisms in terms of computational requirements and processing delay are cryptographic solutions and information protection schemes.

Currently, most common and secure cryptographic schemes are based on complex mathematical problems and high entropy random number generators. These approaches are, however, characterized by large processing delays and a very sparse scalability. Besides, in some occasions, huge redundancies are added, highly reducing the finally provided effective bitrate. Block ciphers, asymmetric keys, secure sessions, and similar solutions based on, for example, elliptic curves are typical solutions presenting these problems [11]. However, lighter cryptographic approaches [12] have been proved to be unsecure against some of the most typical attacks; and (what is more worrying) against modern and future cyber-physical attacks [13]. Then, a totally new and innovative approach is required to communicate access points in the fronthaul and base stations in the backhaul (where large processing resources are available).

Therefore, in this paper we propose a new solution addressing this problem. To provide secure communications in C-RAN architectures we are using a cryptographic solution at physical level, taking advantage of unclonable and non-linear behaviors and effects of optical fiber wires connecting the fronthaul and the backhaul. The proposed technology provides security to communications at physical layer, using only physical signals and mechanisms. Formally, the proposed technique generates an intrinsic Physical Unclonable Function based on resonance structures in optical rings, which produces a chaotic key which is mixed with private information using different techniques such as chaotic masking or modulation. Chaotic schemes may be vulnerable when supported by traditional processing nodes, but chaotic signals generated in optical rings are unclonable, so nobody can replicate their behavior even if they know their design and how the proposed technology operates. As this novel solution works at physical level, delays and bitrate are almost non affected, meeting the requirements of 5G networks.

The structure of the paper is as follows: Section 2 presents the state-of-the-art on information protection mechanisms for 5G networks and current C-RAN solutions. Section 3 describes the main proposal, including the mathematical foundations. Section 4 includes an experimental validation analyzing the performance of the proposed solution. Finally, Section 5 shows the conclusions and future work.

## 2. State of the Art on 5G Security Solutions and C-RAN Solutions

In this section we review the state of the art on C-RAN solutions, focusing on security mechanisms for future 5G networks. Section 2.1 discusses works on general C-RAN technologies and Section 2.2 focuses on 5G security solutions.

### 2.1. C-RAN Architectures, Solutions, and Characteristics

In C-RAN architectures, baseband processing, that is usually distributed among base stations in mobile networks [14], is centralized in a cloud computing center [15]. Two basic elements are identified in C-RAN architectures: the remote radio heads (RRH) and the Baseband processing units (BBU). RRH include all the hardware infrastructure (antennas, reception chains, etc.) required to communicate with the user devices. RRH are deployed in remote places near the users. The network of RRH is named as fronthaul. The fronthaul communicates through dedicated physical links (typically optical links) to the backhaul, a network where BBU are deployed. BBU include all the processing capabilities required to manage communications between the mobile network and/among the users [16]. In 4G scenarios (most common approach nowadays), these BBU are hardware servers located between 20 and 40 km away [17]. This new network design reduces the energy consumption but may be costly in terms of CAPEX (as many large physical servers are needed). Besides, for very dense environments, this approach based on fixed physical server may, eventually, get congested.

Then, in future 5G scenarios, BBU are envisioned to be virtual entities, probably maintained through a small pool of data centers in the cloud [18]. This new approach reduces even more the energy consumption, greatly reduces the CAPEX of mobile networks, and allows a dynamic resource management, so congestion is less probable.

Many different works and applications based on the C-RAN paradigm have been reported, specially for those future engineered systems based on pervasive infrastructures where thousands of devices must communicate [10]. Each one of these applications understand the letter C in a different way: Cloud [17], Centralized processing [19], Cooperative radio [20], Collaborative [21] or Clean [22]. However, most common works try to describe the pending challenges for C-RAN solutions such as new virtualization techniques [23], or strict delay and jitter requirements [24].

Some authors apply C-RAN mechanisms to improve the support of nonuniform traffic and scalability in mobile networks [25]. Techniques such as statistical multiplexing [26] and dynamic resource allocation have been described [27]. Other authors are applying C-RAN paradigm to several different types of networks, such as Wireless Sensor Networks, to reduce energy consumption through the creation of community resource pools [28]. Moreover, some technologies to manage BBU through remote dashboards have been reported [29]. However, most common works on C-RAN applications try to deal with interferences, so the effective bitrate and global delay is improved. Different approaches to reduce the inter-cell interference [30] or employ interference paths constructively [24] may be found.

Finally, different innovative use cases about 5G C-RAN technologies applied to other popular systems have been reported. One of the most common examples is 5G C-RAN for IoT [31], although solutions for Wireless Sensor Networks [32] may be also found.

The security solution proposed in this paper is adequate for any of the previously described technologies and systems, as no computational resources are required. It must be only guaranteed that the fronthaul and the backhaul are connected using optical media.

### 2.2. Security Technologies for C-RAN Architectures

Works on security and 5G networks, as 5G technologies are still under discussion, tend to be very theoretical and abstract. Most articles on this topic present models or logical architectures where security functionalities are identified and described [33], but access networks are managed as a monolithic component where the internal organization is not addressed. In this sense, most common works are only focused on identifying future threats to 5G networks [34,35], such as saturation attacks, TCP-level attacks, resource theft or signaling storms.

Articles describing security solutions in a more detailed manner, are always focused on a particular paradigm or approach to implement future 5G networks. Several authors, then, analyze security solutions for 5G networks when implemented through virtual network functions. A large catalogue of solutions has been reported in this context. From access control mechanisms based on traffic engineering [36], to very specific technologies to prevent IMSI-cracking attacks [37], Denial of Service attacks [38] and identity verification [39]. On the contrary, other works analyze and discuss about enhanced security solutions that are envisioned to be included in future standards by the 3GPPP organization [40].

However, all these works are only addressing security issues in the radio channel between the base station (regardless its internal configuration) and the mobile user. Thus, three topics are usually discussed: user authentication, securitization of wireless links and security policies to be implemented in the user equipment. The specific problems associated to C-RAN architectures are not analyzed.

In fact, a second important group of proposals about security in 5G networks is focused in Radio Access Networks, but only in the radio segment. Lightweight security solutions for resource constrained devices have been reported [41], in particular to guarantee device authentication. Techniques to guarantee service availability and data privacy (through electromagnetic noise or data processing techniques) [42] may be also found. Although in these works, sometimes, access network is considered as two-segment network, solutions are not describing how this structure affects or modifies the technology operation. So, no specific solution to preserve security in communications between those segments is analyzed.

Some authors specifically refer the fronthaul and backhaul networks in their works [43]. Nevertheless, these works are still initial and only discuss about how to integrate security recommendations (for example, from the International Telecommunication Union) in 5G networks. No technological detail is provided.

Security solutions for 5G networks at physical layer are probably the most reported technologies. Although, as previously said, most works are analyzing the security threats, challenges and opportunities [44,45,46]; some specific technologies have been reported. In particular, technologies based on different channel estimators to prevent spoofing attacks in the wireless media have been described [47]. Contrary to these previous solutions, the proposed technique in this paper is focused on the wired media, connecting the fronthaul and the backhaul.

Finally, a sparse collection of security technologies specifically designed for C-RAN architectures may be found. However, in this case, the security policies are still focused either on the mobile service (authentication, impersonation attacks, etc.), or the wireless radio link [48]. Different mechanisms to authenticate user devices in C-RAN architecture have been proposed, but most of them are defined at service level using, for example, XML files and SOAP interfaces [49]. Any case, most numerous works on security and C-RAN architectures are focused on radio channels. Secure handovers [50] and secure downlinks [51,52] in heterogenous wireless access networks have been described. Although all these solutions are designed at message exchange (logical level). Other techniques at radio level for Massive Input Massive Output (MIMO) have been also reported [53,54], although they are focused on communications between user devices and wireless access points. Communications between the fronthaul and the backhaul are, once more, not addressed.

At this point, we are briefly analyzing previous proposals about chaotic intrinsic Physical Unclonable Functions (PUF) in optical media.

No work on this topic applied to 5G networks has been reported. However, some generic proposals about these technologies may be found. In general, chaos in optical and resonant media and structures is analyzed as secondary effect which must be removed to improve the signal quality [55]. Although some practical implementation of resonant structures for intrinsic PUF using optical fibers have been reported [56]. In all these cases, nevertheless, the objective of these PUFs is to create a numerical pseudorandom key to feed a standard encryption scheme [57] or an external and specific modulation component [58]. On the other hand, most practical implementation of chaotic systems in optical communications are based on non-linear effects in laser [59,60], what requires a complex control solution to manipulate optical signals. Contrary to all these works, in our proposal we are taking advantage of two different “random” effects (non-linearities and resonant structures) to provide an entire cryptographic solution at physical level. No algorithm or digital signal processing instrument or software is required. Only physical optical components organized in a specific manner are needed.

## 3. A New Protection Solution for Communications at Physical Layer in C-RAN Architectures

In this Section, the proposed new security technology for C-RAN architectures is described. First, we are discussing about the non-linear phenomena in optical fiber links that may be employed to create chaos at physical level and, then, generate a PUF. The proposed PUF will take advantage of resonant structures to embed the chaotic masking and modulation cryptographic scheme in the optical media (Section 3.2). In Section 3.3, resonant structures and non-linearities in optical fibers are analyzed together, so the produced chaotic signals and masking information is deducted and mathematically defined. Finally (Section 3.4), the whole cryptographic system is presented as an information protection infrastructure.

### 3.1. Non-Linear and Unclonable Effects in Optical Fibers

In standard optical communication systems, linear dynamics are employed to model the signal transmission [61]. This approach is very successful to design effective communication mechanisms, maximizing the link distance and signal quality. Nevertheless, in this approach, all non-linear effects are grouped and modeled as different distortion sources which must be removed or controlled in order to preserve the Signal-to-Noise ratio [61]. However, a very interesting characteristic of all these non-linearities must be considered: they are unclonable.

Non-linearities in optical media are caused by unpredictable and uncontrollable phenomena during manufacturing, the raw material composition and, even, the molecular structure of transmission media. Nowadays, it is impossible to create two optical fiber wires with identical non-linearities, even for manufacturers. An even if the fiber to be replicated is known. These unclonable effects, if exploited, can generate unclonable signals, as response to certain stimuli (or challenges). The resulting functionality is known as intrinsic Physical Unclonable Function (PUF).

In optical media, non-linearities can be stimulated using external nonlinear pumping power sources. However, this approach requires additional active components, energy and complex and precise control mechanisms. Then, in this work, to build the proposed PUF we employ passive non-linear effects which are present in an intrinsic manner in every optical medium: signal modulation due to variations in the refraction index.

In linear models, refraction index in silicon optical fibers η is constant (1). However, due to several uncontrollable effects, this refraction index η changes and varies when the silicon material is under an electromagnetic field $\vec{E}$ (2) with wavelength λ in vacuum. Our silicon material is also absorbent in that wavelength, with a ratio of α dB/m. In the most general case, the function $f_{\eta }$ that governs the behavior of the refraction η index is unknown:

(1) $$\eta =\eta_{0}$$

(2) $$\eta =\eta \left(\vec{E}\right)=f_{\eta }\;\left(\vec{E}\right)$$

Without loss of generality we assume the transmission medium is placed in a cartesian system. We also assume, as in commercial optical fibers, that the media has a cylindrical geometry and it is placed matching the longitudinal axis to *z*-axis. Then, the electromagnetic field $\vec{E}$ may be developed according to cylindrical unitary vectors $\left\{\vec{r},\vec{\phi },\;\vec{z}\right\}$ in that space (3). Moreover, although unknown, the function describing the behavior of refraction index η may be developed using the Taylor’s theorem, and the McLaurin series if the Taylor polynomial is developed around zero (4). To make easier future manipulations, this McLaurin series may be also expressed using the gradient operator ∇:

(3) $$\vec{E}\left(t,\;r,\;z\right)=E_{r}\left(t,\;r,\phi ,\;z\right)\cdot \vec{r}+E_{\phi }\left(t,\;r,\;\phi ,z\right)\cdot \vec{\phi }+E_{z}\left(t,\;r,\phi ,\;z\right)\cdot \vec{z}$$

(4) $$\begin{array}{c} \eta =f_{\eta }\;\left(\vec{E}\right)=\;f_{\eta }\;\left(E_{r},E_{\phi },E_{z}\right)=\\ f_{\eta }\;\left(\vec{0}\right)+\overset{\infty }{\underset{k=0}{\sum }}\frac{1}{k!}\underset{k_{1}+k_{2}+k_{3}=k}{\sum }\left(\begin{array}{c} k\\ k_{1}\cdot k_{2}\cdot k_{3} \end{array}\right)\frac{\partial^{k}f_{\eta }\;\left(\vec{0}\right)}{\partial E_{r}^{k_{1}}\cdot \partial E_{\phi }^{k_{2}}\cdot \partial E_{z}^{k_{3}}}\;\left(E_{r}^{k_{1}}\cdot E_{\phi }^{k_{2}}\;\cdot E_{z}^{k_{3}}\right)\; \end{array}$$

(5) $$\eta =\overset{\infty }{\underset{k=0}{\sum }}\frac{1}{k!}\;{\left(\nabla f_{\eta }\;\left(\vec{0}\right)\cdot \left[E_{r}+E_{\phi }+E_{z}\right]\right)}^{k}\;.$$

This Taylor polynomial may be expressed in a more compact manner, considering new variables $\eta_{k}$ (6)–(7) as:

(6) $$\eta_{k}=\frac{1}{k!}\;{\left(\nabla f_{\eta }\;\left(\vec{0}\right)\right)}^{k},$$

(7) $$\eta =\overset{\infty }{\underset{k=0}{\sum }}\eta_{k}\cdot {\left(E_{r}+E_{\phi }+E_{z}\right)}^{k}\;$$

To make implementable and measurable this infinite series (7), the Taylor polynomial must be truncated, considering a maximum order $N_{max}\;$(8). Some polynomials are well-known and have specific names. For example, if $N_{max}=1$, the resulting phenomena is known as Pockels effect; and the second order them (k=2) is known as Kerr effect:

(8) $$\eta \approx \overset{N_{\max}}{\underset{k=0}{\sum }}\eta_{k}\cdot {\left(E_{r}+E_{\phi }+E_{z}\right)}^{k}\;$$

As the maximum order $N_{max}$ goes up, the real impact of nonlinear effects goes down. However, all of them contribute to generate the final and unclonable signal.

Now, in order to evaluate if the resulting expression defines a nonlinear dynamic, we must find the differential equations describing the behavior of the electromagnetic field in the optical media under study. As commonly assumed, in our optical media there are not electrical charges or external currents. The magnetic permeability is equal to the permeability in the vacuum, $\mu_{0}$. Then, the Maxwell’s equations in the optical media may be simplified (9). $\varepsilon_{0}$ is the vacuum’s electrical permittivity and $\vec{H}$ is the magnetic field generated in the medium:

(9) $$\begin{array}{c} \nabla \;\cdot \left(\eta^{2}\varepsilon_{0}\;\cdot \vec{E}\right)=0,\\ \nabla \times \vec{E}=-\mu_{0}\frac{\partial \vec{H}}{\partial t},\\ \nabla \;\cdot \left(\mu_{0}\cdot \vec{H}\right)=0,\\ \nabla \times \vec{H}=\frac{\partial \left(\eta^{2}\varepsilon_{0}\;\cdot \vec{E}\right)}{\partial t}. \end{array}$$

Calculating the rotational of Faraday’s law, and employing the Ampere’s law, we can obtain a differential equation for obtaining the electrical field (10). The obtained vector equation may be split into two different scalar equations (11). Besides, although energy also propagates in a radial way ($E_{r}\neq 0$), radial signals tend to disappear in the open space, they do not remain within the optical fiber and then they cannot be used for communication purposes. Therefore, we are considering only $E_{z}$ component is employed to communicate and received by the final user:

(10) $$\Delta \vec{E}=-\mu_{0}\varepsilon_{0}\frac{\partial^{2}\left(\eta^{2}\;\cdot \;\vec{E}\right)}{\partial t^{2}},$$

(11) $$\begin{array}{c} \Delta E_{r}=-\mu_{0}\varepsilon_{0}\frac{\partial^{2}\left(\eta^{2}\cdot E_{r}\right)}{\partial t^{2}},\;\\ \Delta E_{\phi }=-\mu_{0}\varepsilon_{0}\frac{\partial^{2}\left(\eta^{2}\cdot E_{\phi }\right)}{\partial t^{2}}\;,\\ \Delta E_{z}=-\mu_{0}\varepsilon_{0}\frac{\partial^{2}\left(\eta^{2}\cdot E_{z}\right)}{\partial t^{2}}. \end{array}$$

With these considerations, and employing the Newton notation for temporal derivatives, the Laplacian operator and the Cauchy product, we can obtain the final expression which should be resolved using the adequate numerical method (12). First, the contour problem should be resolved. This process is not analyzed in this paper, as we are focusing on temporal dynamics employed to generate signals with cryptographic applications. Any case, the contour problem is solved through the continuity conditions in the geometric space r=R, where R is the radix of the optical fiber (optical fibers are open media and fields must be continuous in the transitions with open space).

(12) $$\begin{array}{c} \frac{\partial^{2}E_{i}}{\partial r^{2}}+\frac{1}{r}\frac{\partial E_{i}}{\partial r}+\frac{1}{r^{2}}\frac{\partial^{2}E_{i}}{\partial \phi^{2}}+\frac{\partial^{2}E_{i}}{\partial z^{2}}=-\mu_{0}\varepsilon_{0}\left(2\eta \ddot{\eta }E_{i}+4\eta \;\eta \;˙\dot{E_{i}}+\eta^{2}\ddot{E_{i}}\right),\\ \frac{\partial^{2}E_{i}}{\partial r^{2}}+\frac{1}{r}\frac{\partial E_{i}}{\partial r}+\frac{1}{r^{2}}\frac{\partial^{2}E_{i}}{\partial \phi^{2}}+\frac{\partial^{2}E_{i}}{\partial z^{2}}=\\ =-\mu_{0}\varepsilon_{0}2E_{i}\left[\sum_{k=0}^{N_{\max}}\eta_{k}\cdot k\cdot \left(k-1\right)\cdot {\left(E_{r}+E_{\phi }+E_{z}\right)}^{k-2}\cdot \left(\ddot{E_{r}}+\ddot{E_{\phi }}+\ddot{E_{z}}\right)\;\right]\left[\sum_{k=0}^{N_{\max}}\eta_{k}\cdot {\left(E_{r}+E_{\phi }+E_{z}\right)}^{k}\;\right]-\\ -4\mu_{0}\varepsilon_{0}\left[\sum_{k=0}^{N_{\max}}\eta_{k}\cdot k\cdot {\left(E_{r}+E_{\phi }+E_{z}\right)}^{k-1}\cdot \left(\dot{E_{r}}+\dot{E_{\phi }}+\dot{E_{z}}\right)\;\right]\left[\sum_{k=0}^{N_{\max}}\eta_{k}\cdot {\left(E_{r}+E_{\phi }+E_{z}\right)}^{k}\;\right]\dot{E_{i}}-\\ -\mu_{0}\varepsilon_{0}{\left(\left[\sum_{k=0}^{N_{\max}}\eta_{k}\cdot {\left(E_{r}+E_{\phi }+E_{z}\right)}^{k}\;\right]\right)}^{2}\cdot \ddot{E_{i}},\\ \frac{\partial^{2}E_{i}}{\partial r^{2}}+\frac{1}{r}\frac{\partial E_{i}}{\partial r}+\frac{1}{r^{2}}\frac{\partial^{2}E_{i}}{\partial \phi^{2}}+\frac{\partial^{2}E_{i}}{\partial z^{2}}=\\ =-\mu_{0}\varepsilon_{0}2E_{i}\left(\ddot{E_{r}}+\ddot{E_{\phi }}+\ddot{E_{z}}\right)\left[\sum_{k=0}^{N_{\max}}\sum_{i=0}^{k}\eta_{k-i}\cdot \eta_{k}\cdot k\cdot \left(k-1\right){\left(E_{r}+E_{\phi }+E_{z}\right)}^{2k-2-i}\right]-\\ -4\mu_{0}\varepsilon_{0}\cdot \dot{E_{i}}\left(\dot{E_{r}}+\dot{E_{\phi }}+\dot{E_{z}}\right)\left[\sum_{k=0}^{N_{\max}}\sum_{i=0}^{k}\eta_{k-i}\cdot \eta_{k}\cdot k\cdot {\left(E_{r}+E_{\phi }+E_{z}\right)}^{2k-1-i}\right]-\\ -\mu_{0}\varepsilon_{0}\cdot \ddot{E_{i}}\left[\sum_{k=0}^{N_{\max}}\sum_{i=0}^{k}\eta_{k-i}\cdot \eta_{k}\cdot {\left(E_{r}+E_{\phi }+E_{z}\right)}^{2k-i}\right],\\ i\;\in \left\{r,\phi ,\;z\right\}. \end{array}$$

Then, after solving the contour problem, we obtain an independent function $F\left(t,\;r,\;\phi ,\;z\right)$ for the Laplacian operator (depending on the spatial geometry of the medium), and considering that $\eta_{k}$ coefficients are unknown parameters (whose values depend on manufacturing processes and other uncontrollable phenomena), we can finally obtain a temporal dynamic describing the temporal problem governing the scenario under study (13). In the initial value problem, the initial conditions may be freely selected as they are not controllable in real scenarios. As can be seen, the resulting dynamic presents high-order polynomial terms which show non-linear behavior:

(13) $$\begin{array}{c} F\left(t,\;r,\;\phi ,z\right)=\\ ={\;E}_{i}\left(\ddot{E_{r}}+\ddot{E_{\phi }}+\ddot{E_{z}}\right)\left[\sum_{k=0}^{2N_{\max}-2}a_{k}\cdot {\left(E_{r}+E_{\phi }+E_{z}\right)}^{k}\;\right]+\\ +\dot{E_{i}}\left(\dot{E_{r}}+\dot{E_{\phi }}+\dot{E_{z}}\right)\left[\sum_{k=0}^{2N_{\max}-1}b_{k}\cdot {\left(E_{r}+E_{\phi }+E_{z}\right)}^{k}\;\right]+\\ +\ddot{E_{i}}\left[\sum_{k=0}^{2N_{\max}}c_{k}\cdot {\left(E_{r}+E_{\phi }+E_{z}\right)}^{k}\;\right],\\ i\;\in \left\{r,\phi ,\;z\right\}. \end{array}$$

### 3.2. Resonant Structures in Optical Fibers

The obtained expressions (13) are nonlinear, they define a PUF, but this condition is not enough to guarantee the generation of signals with cryptographic applications (which must be pseudo-random, as chaotic signals). In order to obtain those uncontrollable (chaotic) signals two main requirements must be fulfilled. First, at least three state variables must be defined in the dynamic. The Poincaré-Bendixon theorem stablishes that dynamics with order below three only generate periodic or fixed trajectories [62] (without cryptographic applications). Then, at least three different state variables must be defined. Second, the dynamic must show an unstable behavior. In fact, erratic dynamics must have, at least, one expansion direction to support the uncontrollable behavior of those signals.

**Figure 2:**
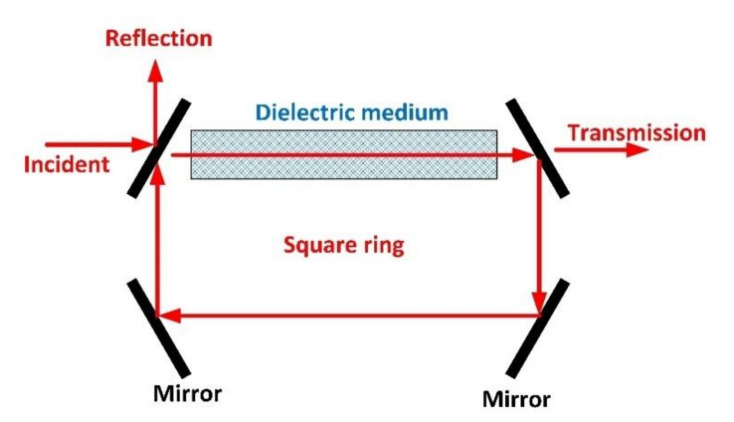
Traditional Akimoto’s system.

Resonant structures may be employed to meet both requirements, especially if a cavity-like structure is created, where the Akimoto instability may be found [56]. The original Akimoto system was made of four mirrors and a dielectric linear medium (see Figure 2), forcing the optical beams to circulate in a square ring. In that system, similar to a resonant cavity, if the dielectric medium presents any nonlinearity (i.e., in any realistic implementation), the electromagnetic signals could be unbounded depending on the excitation signal. Although this infrastructure may support erratic behaviors, it presents two main disadvantages: the construction is complex (mixing two different dielectric materials, mirrors, etc.); and the behavior of the system depends on the excitation signals (what is impractical as communication signals should not be restricted). In this Section we investigate a different cavity-like structure (so the Akimoto instability is present), including new non-idealities such as polarization changes, so the resulting infrastructure supports unstable solutions by its geometry and characteristics, independently from the excitation signal.

**Figure 3:**
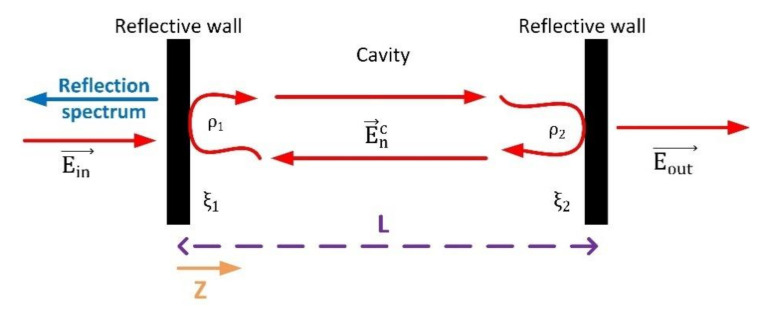
Resonant cavity-like infrastructure.

A traditional cavity-like structure (see Figure 3) is composed of two reflective walls, and a dielectric medium to support the signal transmission. In that structure, three different electrical signals must be considered to study the temporal evolution of the system: the input signal $\vec{E_{in}}$, the signal circulating inside the cavity ${\vec{E}}_{n}^{c}$ (indicating n the number of circulations the signal does inside the cavity) and the output signal $\vec{E_{out}}$. Besides, each reflective wall is characterized by a duple of positive real parameters $\left\{\rho_{i},\xi_{i}\right\}$, where $\rho_{i}$ is the amplitude reflection coefficient and $\xi_{i}$ is the amplitud transmission coefficient. No phase change is induced by reflective walls. It is important to note that $\rho_{i}+\xi_{i}=1$.

If the input signal $\vec{E_{in}}$ is a plane wave and the cavity is ideal, the mathematical expression of ${\vec{E}}_{n}^{c}$ may be easily deducted (14) using the ray theory for electromagnetic waves. In that expression L indicates the length of the cavity (geometric conditions). Now, we are introducing a new realistic non-ideality: interferences inside the cavity. In real optical media, as energy circulates, changes in signal polarization may occur or different transmission modes could be excited. These circulating signals do not match the input signal and, then, it appears a destructive interference. The incident signal and the signal in the cavity cannot be totally added, cause in the interference process some energy is destroyed. To model this situation different strategies may be found but, in this paper, we are using a coupling coefficient γ depending on the wavelength of the optical carrier λ, the first order refraction index $\eta_{0}$, and the geometry of the cavity (15). In our model, this coefficient is, in general, an unknown function and may be modeled using, as previously said, Taylor series (16), and considering a maximum order of $M_{max}$ polynomial terms:

(14) $${\;E\;\to }_{n}^{c}={\;\xi }_{1}\cdot {\left(\rho_{1}\cdot \rho_{2}\right)}^{n}\cdot \vec{E_{\mathrm{in}}}\left(t,\;r,\phi ,2\mathrm{nL}\right),$$

(15) $${\;E\;\to }_{n}^{c}=\;\gamma \left(\lambda ,R,L,\eta_{0}\right)\cdot {\;\xi }_{1}\cdot {\left(\rho_{1}\cdot \rho_{2}\right)}^{n}\cdot \vec{E_{\mathrm{in}}}\left(t,\;r,\phi ,2\mathrm{nL}\right),$$

(16) $$\begin{array}{c} \gamma =\gamma \left(\lambda ,R,L,\eta_{0}\right)=\\ \gamma \left(\vec{0}\right)+\sum_{k=0}^{\infty }\frac{1}{k!}\sum_{k_{1}+k_{2}+k_{3}+k_{4}=k}\left(\begin{array}{c} k\\ k_{1}\cdot k_{2}\cdot k_{3}\cdot k_{4} \end{array}\right)\frac{\partial^{k}\gamma \left(\vec{0}\right)}{\partial \lambda^{k_{1}}\cdot \partial R^{k_{2}}\cdot \partial L^{k_{3}}\cdot \partial \eta_{0}^{k_{4}}}\;\left(\lambda^{k_{1}}\cdot R^{k_{2}}\;\cdot L^{k_{3}}\cdot \eta_{0}^{k_{4}}\right)=\\ =\sum_{k=0}^{\infty }d_{k}\cdot {\left(\lambda +R+L+\eta_{0}\right)}^{k}\approx \sum_{k=0}^{M_{\max}}d_{k}\cdot {\left(\lambda +R+L+\eta_{0}\right)}^{k}. \end{array}$$

Although the reflection spectrum is useful for certain applications, in cryptographic applications we need strong signals, so we are focusing on the output signal $\vec{E_{out}}$. As may be seen, with each circulation in the cavity, a part of the energy is getting out (17), so output optical carrier is time variant (modulated) with a frequency depending on the cavity length, known as resonance frequency $f_{r}$ (18); being $c_{light}$ the light speed in the vacuum:

(17) $$\vec{E_{\mathrm{out}}}\;\left(n,\;r,\phi ,z\right)=\xi_{2}\cdot {\;E\;\to }_{n}^{c}\;$$

(18) $$f_{r}=\frac{c_{\mathrm{light}}}{2L\eta }\approx \frac{c_{\mathrm{light}}}{2{L\eta }_{0}}$$

Any case, standard frequencies in communication signals are much slower than light speed in optical media and, then, than resonance frequencies. Thus, we can consider the variations caused by energy circulating in the cavity as a transitory convergence phenomenon; and we must, then, study the long-term response. This response may be obtained just adding all the partial outputs (19):

(19) $$\begin{array}{ll} \vec{E_{\mathrm{out}}}\;\left(t,\;r,\phi ,z\right) & =\underset{n\to \infty }{\lim}\vec{E_{\mathrm{out}}}\;\left(n,\;r,\phi ,z\right)\\ & =\overset{\infty }{\underset{n=0}{\sum }}\xi_{2}\cdot \xi_{1}\cdot \gamma \left(\lambda ,R,L,\eta_{0}\right)\cdot {\left(\rho_{1}\cdot \rho_{2}\right)}^{n}\cdot \vec{E_{\mathrm{in}}}\left(t,\;r,\phi ,2\mathrm{nL}\right). \end{array}$$

Considering the cylindrical geometry of optical fibers, only components propagating in the *z*-axis could operate in a resonant cavity (20). Besides, as $\rho_{i}$ and $\xi_{i}$ parameters are positive but below the unit, and, because of the energy absorption, the magnitude of electromagnetic waves reduces with each circulation (21). Then, the series may be added, and the result is convergent (22):

(20) $$\begin{array}{c} \vec{E_{\mathrm{out}}}\;\left(t,\;r,\phi ,z\right)=E_{z}^{\mathrm{out}}\;\left(t,\;r,\phi ,z\right)\cdot \vec{z}=\\ =\left(\sum_{n=0}^{\infty }\xi_{2}\cdot \xi_{1}\cdot \gamma \left(\lambda ,R,L,\eta_{0}\right)\cdot {\left(\rho_{1}\cdot \rho_{2}\right)}^{n}\cdot E_{z}^{\mathrm{in}}\left(t,\;r,\phi ,2\mathrm{nL}\right)\;\right)\cdot \vec{z}, \end{array}$$

(21) $$\|E_{z}^{\mathrm{in}}\left(t,\;r,\phi ,2\mathrm{nL}\right)\|<\|{\;E}_{z}^{\mathrm{in}}\left(t,\;r,\phi ,2\left(n+1\right)L\right)\|\forall \;n\;\in \;\mathbb{N}$$

(22) $$E_{z}^{\mathrm{out}}\;\left(t,\;r,\phi ,z\right)={\;\xi }_{2}\cdot \xi_{1}\cdot \gamma \left(\lambda ,R,L,\eta_{0}\right)\cdot \overset{\infty }{\underset{n=0}{\sum }}{\left(\rho_{1}\cdot \rho_{2}\right)}^{n}\cdot E_{z}^{\mathrm{in}}\left(t,\;r,\phi ,2\mathrm{nL}\right)=S<\infty \;.$$

The challenge in the obtained expression (22) is to calculate the components $E_{z}^{in}$ of the electromagnetic waves on the surface of the second reflective wall. Besides, in WDMA (Wavelength-division multiple access) systems the superposition principle should be employed to analyze each optical carrier separately.

### 3.3. Generating Chaotic Resonant Phenomena in Optical Links

**Figure 4:**
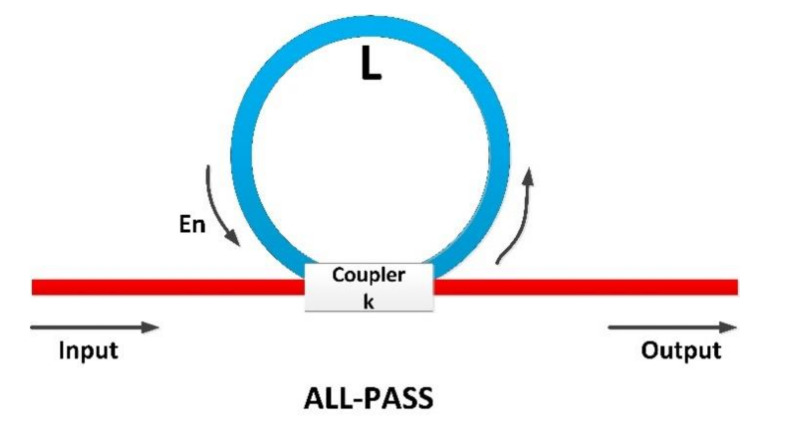
Cavity-like all-pass ring resonator.

The obtained mathematical expression (22) for a cavity-like infrastructure may be easily replicated but using a much more simple and compact solution: an optical ring (see Figure 4). This ring is connected to a main link through a coupler characterized by a coupling parameter κ and δ insertion losses. Then, this all-pass ring represents a resonant cavity satisfying some simple relations (23). This solution, moreover, can be easily integrated into optical links connecting the fronthaul and the backhaul in C-RAN architectures:

(23) $$\begin{array}{c} \rho_{1}=\sqrt{1-\delta },\\ \rho_{2}=\sqrt{1-\kappa },\\ \xi_{1}=1-\delta ,\\ \xi_{2}=\kappa \cdot \sqrt{1-\kappa }. \end{array}$$

Now, as these all-pass rings are made of optical fibers, components $E_{z}^{in}$ may be calculated using the previously obtained non-linear dynamic expression (13). At this point, the problem is well posed and all conditions to guarantee the generation of erratic signals with cryptographic applications are met.

Any case, these conditions are necessary but no sufficient, so we must numerically evaluate if the obtained model can support the expected behavior. Specifically, the referred erratic behavior is mathematically known as chaos. Several different methods to analyze if a non-linear dynamic generates chaos can be employed. Because of its low computational complexity and high visibility, we are using the Lyapunov exponents.

Lyapunov exponents are a measurement that indicates the relative evolution of two trajectories that are as close as desired in the initial point. There is one exponent for each state variable in a dynamic. Chaotic dynamics must diverge in one direction (Lyapunov’s exponent is positive), but they must converge in another one to avoid the hole trajectory to diverge. This equilibrium is, in fact, the origin of chaos. Then, we are evaluating the maximum Lyapunov exponent, analyzing (at the same time) the trajectory is not divergent.

In real applications, although many parameters have been previously introduced, only five are configurable: the wavelength of the optical carrier λ, the length of the ring L, the radix of the optical fiber R, and the characteristics of the coupler, the coupling parameter κ and the insertion losses δ.

More typically, only the length of the ring L and the coupling parameter κ can be freely fixed, as commercial devices present fix values for almost every other parameter. Besides, unknown parameters $a_{k},b_{k},\;c_{k}$ and $d_{k}$ cannot be controlled (as said for Physical Unclonable Functions). Thus, to select values for those parameters we are using a Montecarlo method (several numerical evaluations for different values are performed), and the mean values resulting from these studies are taken the final results. Table 1 represents the selected values for the other parameters. Values for Montecarlo experiment and electromagnetic waves are taken from embedded models in MATLAB software (the software we used for this numerical analysis).

**Figure 5:**
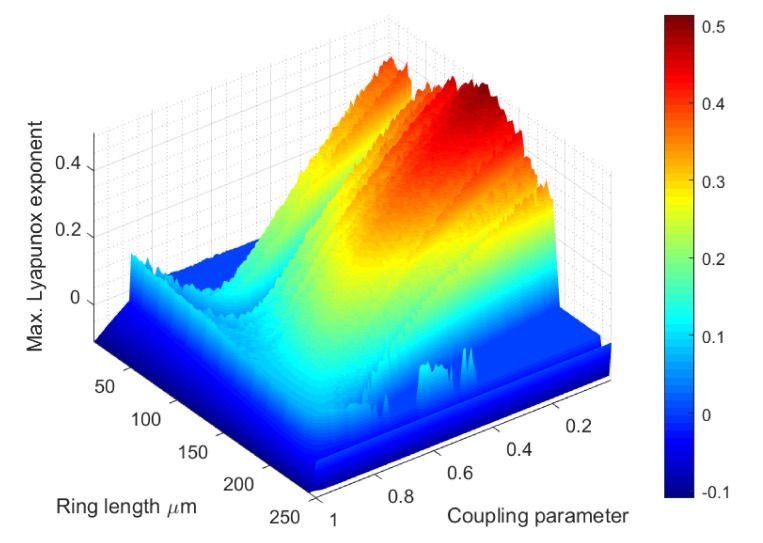
Lyapunov maximum exponent: bidimensional diagram.

Figure 5 shows the obtained results for the maximum Lyapunov exponent and different values of the ring length L and the coupling parameter κ.

As can be seen, the maximum Lyapunov exponent is positive for large areas and combinations of different ring lengths and coupling parameters. Although values are not very high and represent a moderate complex chaos, the obtained results are similar to which generated by other dynamics successfully applied to cryptography (as the Lorenz dynamic) [63]. Thus, the proposed model can be employed in security applications in future C-RAN architectures.

### 3.4. Information Protection Infrastructure

**Figure 6:**
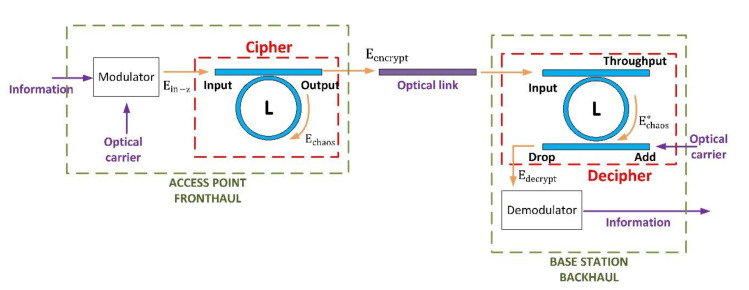
Proposed information protection scheme for C-RAN architectures.

At this point, the proposed solution can generate an unclonable chaotic signal when excited with an optical signal. However, in order to apply the proposed scheme in practical situations, a whole cryptographic mechanism is required. Figure 6 shows that system.

As can be seen, the proposed system includes three different parts: (i) the transmitter, where a cipher at physical level based on the previously described PUF and chaotic signals is included; (ii) an optical fiber link connecting the access point (transmitter) and the base station (receptor) in the C-RAN architecture; and (iii) a receptor, where a complementary resonant ring is included to cancel the chaotic noise in the optical signal and recover the clear information.

As all nonlinearities in the system are additive, and using the superposition principle, we can assume the encrypted signal $E_{encrypt}$ at physical level in the transmitter is the addition of two signals (24): the original information signal $E_{info}$ (associated to linear effects in the optical fiber), and the chaotic masking signal (associated to uncontrollable nonlinear phenomena) $E_{chaos}$:

(24) $$E_{z}^{out}\;\left(t,\;r,\phi ,z\right)=E_{encrypt}\left(t\right)=E_{info}+E_{chaos}$$

The chaotic signal $E_{chaos}$ can only be obtained using the previously described nonlinear dynamic (22), but the information signal $E_{info}$ may be related to the incident wave $E_{in}$ through the traditional linear theory (25), and being α the linear attenuation caused by the energy absorption of materials in the optical fiber:

(25) $$E_{info}=E_{in-z}\cdot e^{-\alpha L}\cdot cos\left(\frac{2\pi }{\lambda }\frac{c_{light}}{\eta_{0}}\;t-\frac{2\pi }{\lambda }L\right)\;.$$

The proposed cipher is an all-pass ring resonator made of optical fiber, which (at the same time) produces the chaotic signal $E_{chaos}$ and mixes it with the clear information $E_{info}$, using a chaotic making scheme (where the chaotic signal hides the original information). This encryption (masking) mechanism is supported by the physical structure of the ring, and its electromagnetic response when excited with the optical carrier. No computational infrastructure of resource is needed. All operations are performed at physical level.

Then, the access point in the fronthaul and the base station in the backhaul are connected through an optical link. This link is connected to a receptor where the chaotic masking process is reversed (decryption). To do that, the decipher includes a new ring resonator, which must be built using the exact same optical fiber than the ring in the cipher (to ensure both have the same nonlinearities). This new ring, however, follows an add-drop paradigm. The encrypted signal $E_{encrypt}$ is introduced in the “input” port. This ring is, besides, excited in the “add” port using an “empty” optical carrier (26) in the same wavelength than the encrypted signal. Then, if the optical fiber is exactly the same than the one employed in the cipher, the same chaotic signal $E_{chaos}^{*}$ will be generated in the ring (27). Couplers in the transmitter and receptor to be as similar as possible. This chaotic signal is added to the encrypted signal in the “throughput” port (28), but it is subtracted in the “drop” port (this result may be easily deducted using the ray theory for optical waves) (29):

(26) $$E_{\mathrm{add}}\;\left(t,\;r,\phi ,z\right)=\;\cos\left(\frac{2\pi }{\lambda }\frac{c_{\mathrm{light}}}{\eta_{0}}t\right)\;,$$

(27) $$E_{z}^{{\mathrm{out}}^{*}}\left(t,\;r,\phi ,z\right)=E_{\mathrm{chaos}}^{*}+{\;E}_{\mathrm{add}}={\;E}_{\mathrm{chaos}}+E_{\mathrm{add}},$$

(28) $$E_{\mathrm{through}}\;\left(t,\;r,\phi ,z\right)={\;E}_{z}^{{\mathrm{out}}^{*}}+E_{\mathrm{encrypt}},$$

(29) $$\begin{array}{c} E_{\mathrm{drop}}\;\left(t,\;r,\phi ,z\right)={\;E}_{\mathrm{encrypt}}-E_{z}^{{\mathrm{out}}^{*}}={\;E}_{\mathrm{info}}+{\;E}_{\mathrm{chaos}}-\left(E_{\mathrm{chaos}}+E_{\mathrm{add}}\right)=\\ ={\;E}_{\mathrm{info}}+\cos\left(\frac{2\pi }{\lambda }\frac{c_{\mathrm{light}}}{\eta_{0}}t\right). \end{array}$$

The decrypted signal $E_{decrypt}$ is, then, directly obtained (the chaotic masking is removed). And only a peak of optical power in the frequency of the carrier is added (30). However, this spurious energy will be removed in the demodulation and detection device. The original private information is then recovered in the base station with no computational cost (only physical phenomena are employed). Some distortion caused by transmission degradation may appear, but how resilient to these effects is the proposed scheme will be later evaluated. Furthermore, if chaotic signals are weak in the employed optical fiber, they can be always strengthen using optical amplifiers with no computational consumption:

(30) $$E_{\mathrm{decrypt}}={\;E}_{\mathrm{drop}}={\;E}_{\mathrm{info}}+\cos\left(\frac{2\pi }{\lambda }\frac{c_{\mathrm{light}}}{\eta_{0}}t\right)$$

## 4. Experimental Validation and Results

In order to evaluate the performance of the proposed technology, we are carrying out a validation composed of two experiments. The first experiment was focused on the performance of the solution as security and information protection technology. The second experiment was focused on analyzing the proposed technique from the telecommunication engineering point of view.

All these experiments were implemented using a simulation scenario. The selected simulation scenario represented a quite realistic application scenario of 5G networks, although the number of final devices, access point or base station does not affect the described technology. Then, a fronthaul composed of one hundred access points and a backhaul compose of twenty base station is considered (similar values may be currently found in metropolitan areas). The distance between both elements was fixed to 3 km (a standard value in real scenarios). Besides, the access points are provided with the proposed cipher and the base station with the corresponding decipher. All the simulations were performed using the MATLAB 2019a software, where specific models for optical communications are embedded. All simulations were performed using a Linux architecture (Linux 16.04 LTS) with the following hardware characteristics: Dell R540 Rack 2U, 96 GB RAM, two processors Intel Xeon Silver 4114 2.2G, HD 2TB SATA 7,2K rpm. Optical communications were designed to work on the second optical window. Table 2 describes the values for all relevant parameters in the proposed simulations [64]. Parameters that are not included in Table 2 are employed as independent or control variables.

Modulation in the modeled C-RAN architecture was OOK (On-Off Keying) modulation. The frequency of information to be transmitted was 150 MHz, to meet the requirements and characteristic of future 5G networks are envisioned nowadays.

Using this simulation scenario and software, two experiments were designed. The first one evaluates how strong is the proposed cryptographic scheme using two techniques: a heuristic technique comparing the original and the encrypted signal in the temporal and the frequency domains; and an evaluation of the mutual information indicator between the raw information signal and the encrypted signal. This second technique was based on several different simulation scenarios where different chaotic signals were induced varying the ring length and the coupling parameters. Each chaotic signal was represented by its maximum Lyapunov exponent. Final results were calculated with the average of twelve different simulations for each situation, and the average for all considered access points and bases stations. Besides, each simulation represented an operation time of seventy-two (72) hours. To enable the calculation of the mutual information, original and encrypted signals were quantified using symbol composed of twenty (20) bits. Then, 2048 different values could be defined.

The second experiment was focused on the performance of the proposed solution from the telecommunication engineering point of view. Two basic and relevant indicators were evaluated: the Bit Error Rate (BER) and the relative error (or error dispersion) between the original and the decrypted signals at physical level. The experiment is repeated for different types of chaos and when differences in the coupler are observed. As in the first experiment, final results were calculated with the average of twelve different simulations for each situation (and the average for all considered access points and bases stations), and each simulation represented 72 h of operation.

**Figure 7:**
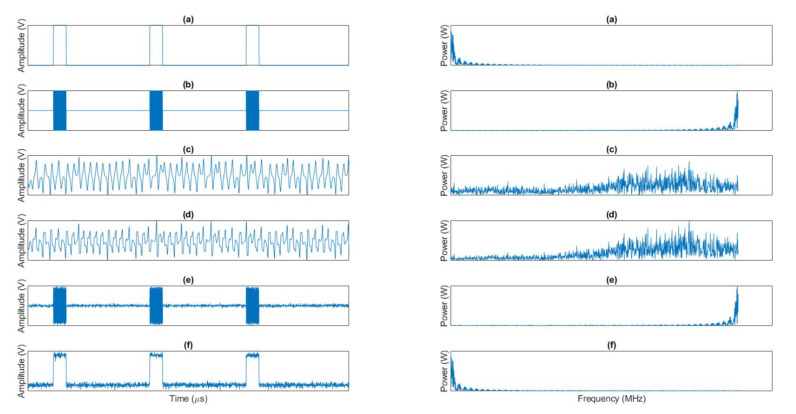
Comparison among the most relevant signals in the time (left) and frequency (right) domain.

Figure 7 shows a comparison between most relevant signals in the proposed information protection scheme in both domains: temporal and frequency. For clarity, only a short time and most relevant frequencies are showed. Besides, all amplitude, time and frequency scales have been normalized, as (in this case) we are only focusing on waveforms.

As can be seen, original information (a) is recovered in a high-quality manner (f), as only some additive noise (probably some residual chaotic signal) is affecting the received information. Despite this fact, in the optical link, the chaotic signal (c) masks and hides the modulated information signal (b), so the encrypted signal (d) is totally erratic and no pattern may be easily detected. In the frequency spectrum the phenomenon is similar, as chaotic signals presents large and sparse spectrum being able to hide even signals in 5G broadband communications.

**Figure 8:**
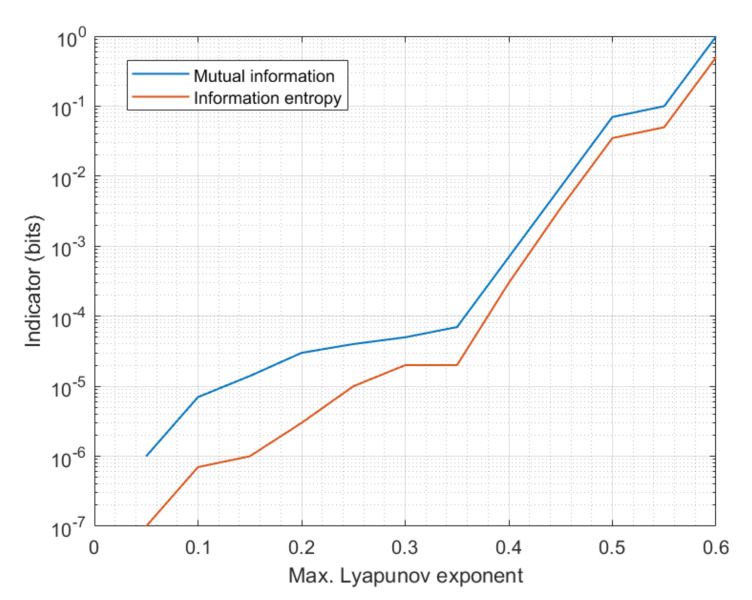
Mutual information between the encrypted and clear signals.

However, in order to analyze if sophisticated techniques could find any residual private information in the chaotic masked signal, we are obtaining the mutual information between the modulated signal and the chaotic signal for different types of chaos. Figure 8 shows the obtained results. Mutual information has been normalized in order to make independent the results from the symbol scheme selected to quantify signals. To complement this result, the entropy (according to Shannon’s definition) of the chaotic signal is also obtained.

As can be seen, the normalized mutual information gets closer to the unit as the Lyapunov exponent goes up and the complexity of generated chaos grows up. That means that, as chaos gets more complex, the encrypted signal does not contain any amount of private information and, then, no technique (although very exhaustive) can break the proposed encryption. This evolution is caused by the higher entropy of chaotic signals as maximum Lyapunov exponent goes up. Figure 8 also shows how entropy tends to 0.5 bits (maximum entropy and randomness) as Lyapunov exponents grow.

**Figure 9:**
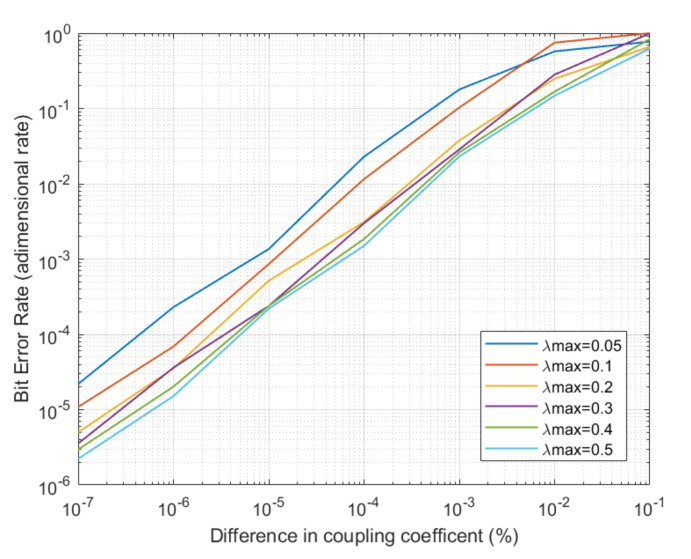
Evolution of BER for different system configurations.

**Figure 10:**
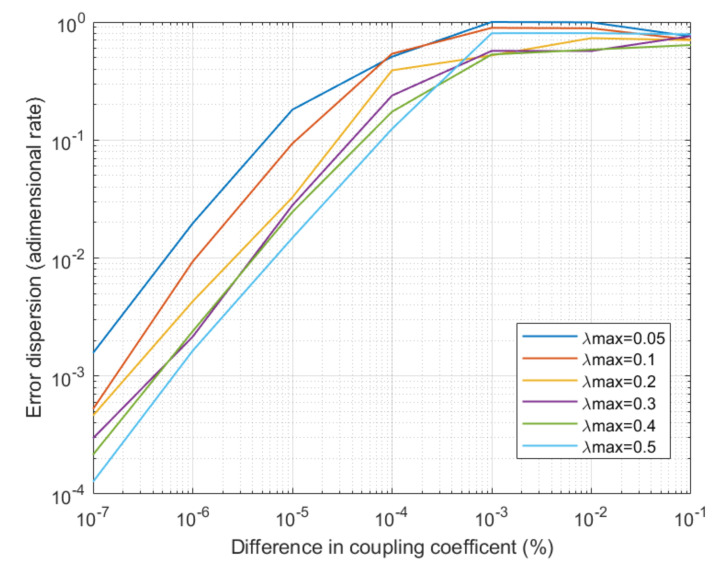
Evolution of error dispersion for different system configurations.

As stated in Section 3, couplers in the receptor and transmitter must be selected as similar as possible. However, it may be very complicated or impossible to obtain two identical devices. Thus, a key question to answer is how differences in those devices affect the system performance. The experiment is also repeated for different types of chaos. Figure 9 and Figure 10 show the obtained results about this experiment.

The obtained results show that BER up to $10^{-5}$ may be achieved for medium complexity chaos when couplers are exactly the same device, although error bursts may appear (with a duration of some milliseconds). These bursts, any case, do not affect the long-term performance. The relative error (or error dispersion) may be also low, reaching a value of $10^{-3}$ for medium complexity chaos signals. If differences are observed between couplers in the transmitter and the receptor, the BER and error dispersion grows exponentially as differences between couplers are higher. This error rates are near 100% when coupling parameters are different in more than 10%, regardless the type of chaos being generated. Besides, it can be observed how error dispersion goes up faster than BER, as bit recognition mechanisms are prepared to tolerate even high levels of distortion. In a standard scenario with differences around 0.0001% in the coupling parameters (a quite real value), the BER is around $10^{-4}$ and error dispersion around $10^{-2}$.

## 5. Conclusions and Future Works

In this paper, it is proposed a novel information protection scheme for C-RAN architectures based on resonant phenomena in optical fibers communicating the fronthaul and backhaul in 5G networks. Resonant structures (specifically optical rings) and physical nonlinearities generate an unclonable chaotic signal which may encrypt and hide at physical level every communication stream in a very efficient manner. The proposed model includes two novelties: a more complex representation of refraction indices in optical fibers (affected by uncontrollable nonlinearities), and the consideration of random and unknown destructive interferences in the optical ring.

The resulting system is generating a chaotic signal with a similar complexity to other existing chaotic dynamics in the state of the art for cryptographic applications.

The experimental validation shows the proposed solution totally masks the private information being protected, in terms of waveform and information. Besides, in real scenarios, a Bit Error Rate around $10^{-4}$ may be achieved. In future works, the proposed mechanism will be deployed using real commercial components, in order to analyze its performance in realistic applications.

## Figures and Tables

**Table 1:** Selected values for the Lyapunov numerical study.

Parameter	Value	Comments
δ	0.1 dB	Common commercial value
λ	1500 nm	Common value
$\vec{E_{\mathrm{in}}}$	$cos\left(\frac{2\pi }{\lambda }c_{light}\cdot t\right)$	Elemental harmonic signal
R	5 μm	Common commercial value

**Table 2:** Selected values for the experimental validation.

Parameter	Value	Comments
δ	0.1 dB	Common commercial value
λ	1310 nm	Second optical window
$\vec{E_{\mathrm{in}}}$	-	Random sequences of bits
R	5 μm	Common commercial value
